# Evaluation of a Cardiopulmonary Resuscitation Video Decision Aid for Pet Owners

**DOI:** 10.1111/vec.70070

**Published:** 2025-12-12

**Authors:** Katherine M. Gane, Lotta Wahlden, Benjamin W. N. Fayers, Tim H. Sparks, Emily Thomas

**Affiliations:** ^1^ Dick White Referrals Cambridgeshire UK; ^2^ Waltham Petcare Science Institute Melton Mowbray UK

**Keywords:** client communication, decision making, decision support tool, patient choice, pet owner education

## Abstract

**Objective:**

To evaluate the impact of a Cardiopulmonary Resuscitation Video Decision Aid (CPR‐VDA) on pet owners’ resuscitation preferences, decisional confidence, conflict, and knowledge of CPR.

**Design:**

Prospective, interventional survey study with data collected from January to March 2024. Owners of pets presenting through the Emergency Department completed a preintervention questionnaire. They then viewed a 7‐min CPR‐VDA and completed a postintervention questionnaire. Changes between pre‐ and postintervention scores were analyzed using Wilcoxon signed rank tests.

**Setting:**

Private referral hospital

**Subjects:**

Seventy‐eight pet owners

**Interventions:**

Viewing of a CPR‐VDA

**Measurements and Main Results:**

The preintervention questionnaire assessed pet owner demographics, resuscitation preferences, decisional confidence and conflict, and prior knowledge about CPR. The postintervention questionnaire reassessed resuscitation preferences, decisional confidence and conflict, and CPR knowledge, with additional questions evaluating owner’ perceptions of the CPR‐VDA. Preintervention, 56 of 78 (72%) participants expressed a preference for “resuscitation” of their pet, five of 78 (6%) opted for “do not attempt resuscitation,” and 17 of 78 (22%) were “not sure.” Most participants (56/78 [72%]) did not change their resuscitation preference following the intervention. Participants felt more confident in their decision after watching the CPR‐VDA (*p* < 0.001). There was no difference in how conflicted they felt making this decision (median score on a rating scale of 1–5 was 2.25 preintervention and 2.42 postintervention; *p* = 0.192). Their knowledge of CPR improved (median correct answers increasing from 5.5/9 to 9/9; *p* < 0.001). Most participants rated the video “good” or “excellent” (72/78 [92%]), found the video helpful (62/78 [79%]), and were likely to recommend it to other pet owners (72/78 [92%]).

**Conclusions:**

The use of a CPR‐VDA improved pet owner knowledge of CPR and increased their confidence in decision making. The CPR‐VDA was well received by pet owners and may be a useful tool to facilitate discussions regarding this topic.

**Abbreviations**
CPR‐VDA: Cardiopulmonary Resuscitation Video Decision Aid
IPDAS: International Patient Decision Aids Standards
RECOVER: Reassessment Campaign on Veterinary Resuscitation

## 1 Introduction

Decision aids are written or audiovisual tools that provide evidence‐based information about treatment options, benefits, risks, and outcomes, and encourage active participation from the patient to help communicate their preferences with healthcare providers [1]. They are often used in human medicine and can make it easier for patients and healthcare professionals to discuss treatment options [2]. People exposed to decision aids felt more knowledgeable, had a better match between values and choices, and showed a reduction in indecision and regret that leads to decisional conflict [2]. Compared with verbal description, video decision aids have been shown to reduce uncertainty in decision making and increase preferences for more comfort‐focused medical care [3].

Cardiopulmonary resuscitation (CPR) can be a difficult and sensitive subject for discussion with pet owners. The reasons for this are likely multifactorial, with a recent survey in the United Kingdom showing that veterinary professionals face a number of challenges when discussing CPR with pet owners, including fear of causing upset, lack of time, and unrealistic owner expectations [4]. Previous studies have demonstrated lack of pet owner knowledge of CPR procedures, alongside unrealistic perception of outcomes. Pet owners have been shown to overestimate survival to discharge rates between 39% and 45% following CPR [5, 6]. In contrast, actual survival to discharge rates reported in a recent study were 7% and 19% for dogs and cats, respectively [7]. Owners’ unrealistic perceptions may contribute to the challenging nature of these discussions. Nevertheless, pet owners would like to be better educated in CPR and have prognosis, possible complications, and costs explained to them in order to make an informed decision on their pet's resuscitation status [4, 5, 6].

In human health care, these issues are mitigated by providing written or video‐based information on CPR prior to or outside of the consultation [8]. After watching a short video on CPR, people felt better informed about their own resuscitation choices and were more likely to discuss their CPR preferences with their healthcare providers and receive medical care that was aligned with their stated wishes [9]. Interestingly, patients with more accurate perceptions of CPR survival rates were more likely to refuse CPR for themselves [10]. While this has not been explored in veterinary medicine, it seems reasonable to extrapolate that an improved understanding of CPR procedures and outcomes may impact owner resuscitation preferences for their pet and, at a minimum, that provision of accurate information is important when obtaining informed consent for CPR or do‐not‐resuscitate orders in small animals.

The aim of this study was to evaluate the impact of a Cardiopulmonary Resuscitation Video Decision Aid (CPR‐VDA) on pet owners’ resuscitation preferences, decisional confidence and conflict, and knowledge of CPR. Our hypotheses were that pet owner knowledge and confidence would increase when the CPR‐VDA was used, that decisional conflict would decrease, and that more pet owners would opt for “do‐not‐resuscitate” status after watching the CPR‐VDA.

## 2 Materials and Methods

### 2.1 Study Design and Inclusion Criteria

Owners of pets presenting through the Emergency Department of a private referral hospital were enrolled between January 1 and March 1, 2024. Exclusion criteria were owners of pets presenting to other departments and owners whose pet was deemed by the attending clinician to be at immediate risk of cardiopulmonary arrest. Additionally, pet owners were excluded if a study investigator was not available at that time to enroll them in the study, or if the only study investigator available was also the primary clinician for their pet. Participation in the study was entirely voluntary, and pet owners were given an information sheet and consent form to read and sign prior to participation. Ethical approval was granted prior to data collection by the Royal College of Veterinary Surgeons (RCVS) Ethical Review Panel (reference 2023–095).

Study participants were asked to complete a short questionnaire, watch the CPR‐VDA (intervention), and then complete another short questionnaire. Questionnaires were completed via an online survey tool^1^ using a handheld tablet (provided as Supporting Information). The preintervention questionnaire contained single‐answer, multiple‐choice, Likert‐scale, and free‐text questions relating to participants’ demographics (*n* = 4 questions), resuscitation preferences (*n* = 1), how confident participants felt about their resuscitation preference (*n* = 1), how conflicted they felt in taking this decision (*n* = 1), and prior knowledge about CPR (*n* = 9).

The postintervention questionnaire repeated the questions relating to resuscitation preferences, decisional confidence and conflict, and knowledge about CPR, alongside additional questions regarding the owners’ perceptions of the CPR‐VDA (*n* = 7), with an opportunity to leave free‐text comments about the video or survey.

Correct answers to knowledge assessment questions were each given a score of 1 for a possible total of nine correct answers. Decisional confidence and conflict were assessed using 5‐point Likert scales.

The 7‐min CPR‐VDA demonstrated CPR being performed on a canine mannequin and described CPR interventions, including intubation, chest compressions, and drug administration alongside alternative options such as euthanasia. The interventions shown followed the Reassessment Campaign on Veterinary Resuscitation (RECOVER) guidelines current at the time of data collection [11]. Survival statistics for both human and veterinary CPR were conveyed via pictographs [12, 13, 14]. The CPR‐VDA underwent a systematic development process as recommended by the International Patient Decision Aids Standards (IPDAS) and was assessed for accuracy by a Diplomate of the American College of Veterinary Emergency and Critical Care and a RECOVER Certified ALS Rescuer. Information was provided in plain language with both audio and video to aid pet owner understanding.

During study design, the survey and video were piloted with pet owners. The survey and video were found to be clear and easy to understand, and based on this pilot, a completion time of approximately 15 min was estimated for study completion (including watching the video and answering both questionnaires). Pet owner enrollment was carried out during patient triage by one of the study investigators. Study participation took place in a private space (consultation room), and patient triage was performed in a separate room (Emergency Room) to minimize distractions. The study investigator was not involved with the triage or treatment of the owner's pet. When two owners presented with one pet, both were given the option of participating separately. Following enrollment, the study investigator opened the preintervention questionnaire on the tablet to get participants started and then left the consultation room to offer the participant privacy while completing the study and to reduce social desirability bias by the presence of the investigator [15]. Participants were given clear verbal and written instructions to watch the video before completing the postintervention questionnaire. In addition, after completion of the preintervention questionnaire, a message was displayed on the tablet requesting owners to watch the video and press “next” after watching it to access the postintervention questionnaire. The study investigator remained nearby the outside of the consultation room to answer any questions during study completion.

After study participation, the pet's primary clinician proceeded with a consultation as normal including separate confirmation of the owner's CPR preference for their pet to ensure that the final decision reflected clinical information specific to their pet. Asking pet owners for a resuscitation status is standard practice for all patients admitted by the Emergency & Critical Care Service at the authors’ hospital.

### 2.2 Sample Size

The chosen sample size was influenced by Cohen's [16] concept of a medium effect size, but without knowledge of the level of pre–post correlation in responses. Defaulting to a two‐sample test (rather than a paired test) and allowing for a 15% uplift in sample size to accommodate nonparametric testing suggested a minimum sample size of 74 paired responses at a significance level of 5% and a power of 80%.

### 2.3 Data Analysis

Responses were downloaded using a commercial spreadsheet software program^2^ for analysis. Pet owner demographics are reported as median (range) for continuous and ordinal data, and as frequency and percentages for binary and categorical data. Key aspects of demographics were compared to initial CPR choice, change in CPR choice, and pre‐knowledge of CPR using rank correlation for ordinal–ordinal comparisons or using Kruskal–Wallis tests adjusted for ties for ordinal–categorical comparisons. Changes between pre‐ and postintervention responses were analyzed by Wilcoxon signed rank tests for ordinal data. Analysis was undertaken in a statistical software program^3^. Significance was considered as *p* < 0.05.

Quantitative content analysis was performed on free‐text data. Free‐text responses were reviewed by one author and assigned into predefined categories (Table 1). The most common responses were reported, and individual comments that were representative of general comments under that category were chosen for quotation in the manuscript.

**TABLE 1:** Summary of pet owner free‐text comments after viewing a Cardiopulmonary Resuscitation Video Decision Aid for their pet, including problems or distractions experienced while viewing the video.

Question	Categories *n*/*N* (%)	Comment example
Please leave any comments you may have about the video or survey if you wish	Informative 8/78 (10%)	Excellent informative video
		I think this video or similar videos should be available for all owners, for some people who are unaware completely of what it entails this is very informative
	Alluding to reason for CPA may affect resuscitation decision 4/78 (5%)	All depends on the condition that is being treated
		…I probably have a different answer to whether or not I would request CPR depending on the context of when CPR might be given
	Suggested improvements 6/78 (8%)	More images and videos would be helpful
		A little more detail/facts wouldn't have caused an overload of information
		A further breakdown of the decision‐making process with examples would be useful in my view
		No mention of risks of CPR
Did you experience any problems or distractions while viewing the video?	IT problems 2/78 (3%)	Had to ask for help to play video but I'm not good with IT
	Noise distractions 1/78 (1%)	Vacuuming outside consult room

Abbreviations: CPA, cardiopulmonary arrest; IT, information technology; *n*, number of responses to the question; *N*, total number of questionnaires completed.

## 3 Results

Ninety‐nine pet owners were approached for study inclusion. Nineteen (19/99 [19%]) declined participation, with the most common reasons being “too upset” (6/19 [32%]) or “stressed” (5/19 [26%]). Other reasons included being “overwhelmed” (2/19 [10%]) or busy completing other paperwork (1/19 [5%]). Another five of 19 [26%] participants gave no reason. One was excluded as their pet was deemed to be at imminent risk of cardiac arrest (1/19 [5%]), and one of 19 (5%) was withdrawn due to a video malfunction. As a result, 78 were included in the study.

The median age of participants was 48 years (16–78) with two participants disclosing their ages as 5 and 8, respectively. These participants were excluded from age analysis due to obvious typographical error, as recruiting investigators confirmed that no young children were enrolled. The majority of participants were female (57/78 [73%]), with the remaining 19/78 (24%) being male and two of 78 (3%) identifying as other genders. When asked to disclose their highest level of education, most participants had completed a vocational qualification (21/78 [27%]) or postgraduate degree (23/78 [29%]). When asked how important religion was to participants, the majority (46/77 [60%]) answered “not at all important,” with only two of 77 (3%) stating religion as “very important.” There was no association between age, education, and religious beliefs and any of initial CPR preference, change in CPR preference, or pre‐knowledge of CPR (Table 2).

**TABLE 2:** Influence of pet owner demographics on their resuscitation preference for their pet, change in resuscitation preference following the viewing of a CPR Video Decision Aid, and pre‐existing knowledge of CPR prior to viewing the decision aid. Statistical tests, derived from *n* = 75–78 responses, based on either Spearman rank correlation (*r*_s_) or Kruskal–Wallis (*H*), as appropriate. Significance was considered as *p* < 0.05.

	Resuscitation preference		Change in resuscitation preference		Pre‐knowledge of CPR	
	*r*_s_	*p*	*r*_s_	*p*	*r*_s_	*p*
Age	−0.16	0.165	−0.07	0.530	−0.04	0.218
Importance of religion	−0.12	0.277	−0.01	0.936	−0.14	0.230

	*H*	*p*	*H*	*p*	*H*	*p*
Education	1.95	0.582	2.73	0.436	5.41	0.144

When asked “have you previously discussed CPR for this pet,” most participants answered no (71/78 [91%]), six of 78 (8%) had previously discussed this for their pet, and one of 78 (1%) was not sure.

**FIGURE 1:**
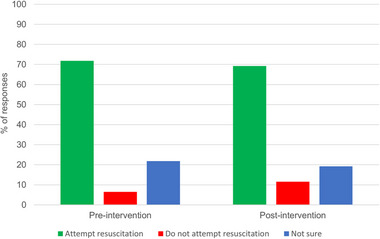
Bar chart showing pet owner resuscitation preferences for their pet before and after watching a CPR video decision aid (intervention). Data are presented as percentages of total responses. Left/green bar, “Attempt resuscitation”; middle/red bar, “Do not attempt resuscitation”; blue/right bar, “Not sure.”

**FIGURE 2:**
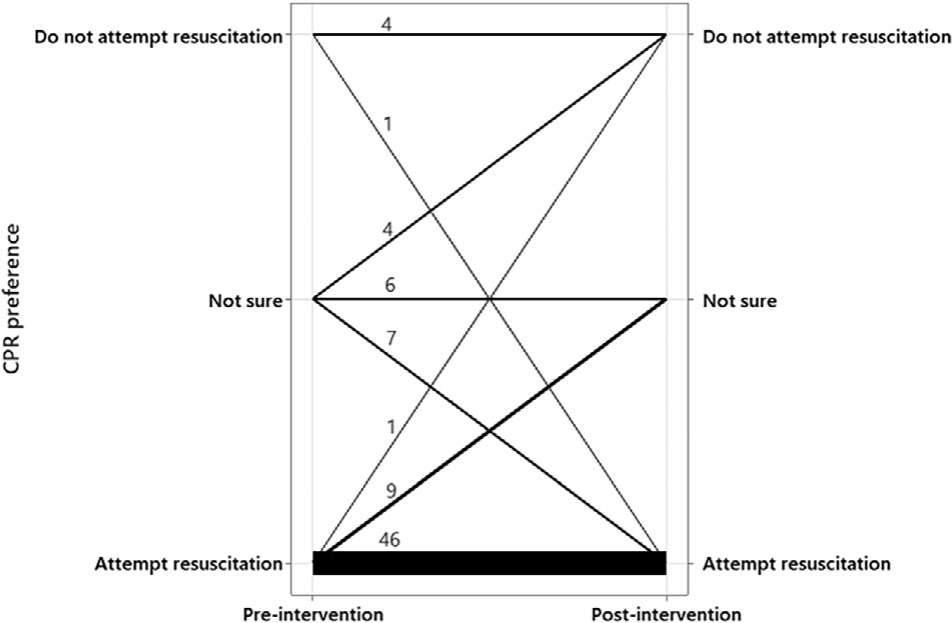
Diagram showing pet owners’ resuscitation preferences for their pet before and after watching a CPR video decision aid (*n* = 78). The thickness of the line is proportional to number of responses, with the absolute numbers appearing above each connecting line.

When asked in the preintervention questionnaire what their resuscitation preference would be for their pet, 56 of 78 (72%) opted for “resuscitation,” five of 78 (6%) opted for “do not attempt resuscitation,” and 17 of 78 (22%) were “not sure.” Most participants (56/78 [72%]) did not change their resuscitation preference following the intervention, but 22 of 78 (28%) did change preference (Figure 1). Change in preference did not appear to be directional, with nine of 78 (12%) changing from resuscitation to “not sure” and seven of 78 (9%) changing from “not sure” to resuscitation (Figure 2).

**FIGURE 3:**
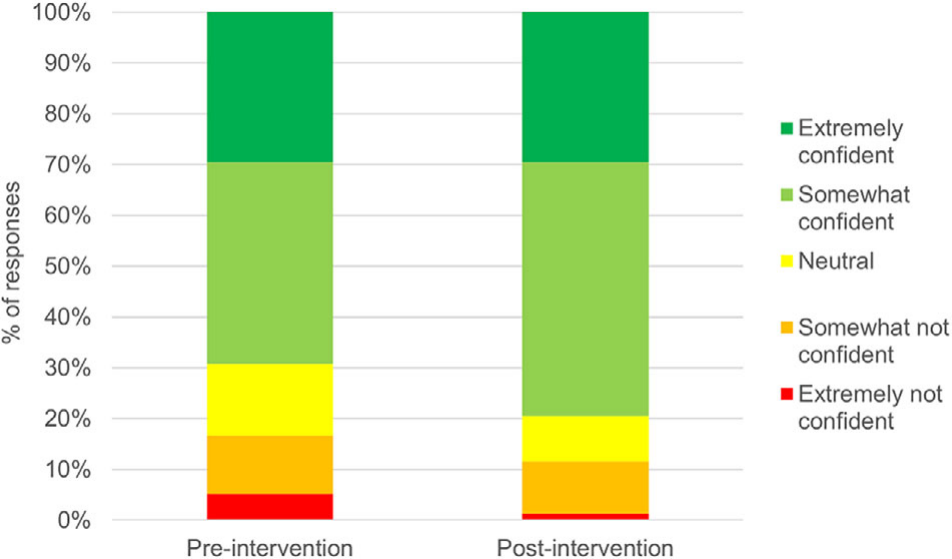
Bar chart showing pet owners’ levels of confidence pre‐ and post‐CPR informational video intervention in response to the question “how confident do you feel making a decision about CPR for your pet?” Data are represented as percentages. Extremely confident, dark green/top section of each bar; Somewhat confident, light green/second‐from‐top section of each bar; Neutral, yellow/middle section of each bar; Somewhat not confident, second–from–bottom section of each bar; Extremely not confident, bottom section of each bar.

Participant answers to the preintervention question “how confident do you feel making a decision about CPR for your pet” showed that the majority felt “extremely confident” (23/78 [29%]) or “somewhat confident” (31/78 [40%]), and fewer participants felt “neutral” (11/78 [14%]), “somewhat not confident” (9/78 [12%]), or “extremely not confident” (4/78 [5%]). Participants felt more confident in their resuscitation decision after watching the CPR‐VDA (*p* < 0.001) (Figure 3), with an increased number of participants feeling “somewhat confident” (39/78 [50%]) and fewer participants feeling “neutral” (7/78 [9%]), “somewhat not confident” (8/78) [10%]), or “extremely not confident” (1/78 [1%]). Participants were asked to score their decisional conflict regarding resuscitation decisions for their pet on a scale of 1–5. A score of 1 reflected “no conflict” and a score of 5 indicated “high conflict” felt making this decision. Preintervention, the median score was 2.25 (1–5), and postintervention, it was 2.42 (1–5). There was no difference (*p* = 0.192) in how conflicted participants felt making this decision pre‐ and post‐video intervention.

**FIGURE 4:**
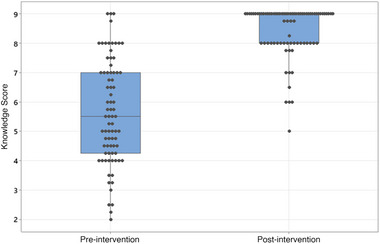
Box and whisker plot representing pet owners’ CPR knowledge scores pre‐ and post‐video intervention as assessed by nine multiple choice questions relating to CPR. The box represents the first and third quartile, and the central line represents the median. The outer extent of the whiskers represents the maximum and minimum knowledge score by individual participants.

Regarding the knowledge assessment questions, the median number of correct answers before intervention was 5.5/9 (range 2/9–9/9) with an increase to 9/9 (5/9–9/9) after intervention (*p* < 0.001; Figure 4). The greatest knowledge impact after the CPR‐VDA related to pet survival. Preintervention, only 30 of 78 (38%) participants correctly identified that few pets survive to discharge after cardiopulmonary arrest due to an underlying disease process. This increased to 75 of 78 (96%) following the video intervention. Similarly, preintervention, 18 of 78 (23%) people correctly answered that almost half of pets sustaining cardiopulmonary arrest under anesthesia survive to discharge. Following the intervention, 58 of 78 (74%) correctly answered this. Participants had a clearer understanding of the definition of CPR and the interventions it entails. Preintervention, 70 of 78 (90%) pet owners agreed with the statement that “CPR is a medical procedure that is performed on pets whose heart has stopped beating in attempt to restart their heart.” This increased to 77 of 78 (99%) after intervention. Preintervention, 53 of 78 (68%) of participants correctly identified that CPR includes interventions such as intubation, compressions, medications, and defibrillation. This increased to 75 of 78 (96%) postintervention. After intervention, 75 of 78 (96%) understood that pets would need ongoing care following successful CPR compared to 57 of 78 (73%) before intervention.

Answers to questions relating to acceptability of the CPR‐VDA are summarized in Tables 1 and 3. Overall rating of the CPR‐VDA was predominantly “good” to “excellent” (72/78 [92%]), with participants mainly rating it as clear, balanced, and providing the right amount of information. Overall, most participants found the video helpful for making decisions about CPR and the majority were likely to recommend it to other pet owners (72/78 [92%]) (Table 3). At the end of the questionnaire, participants could add free‐text comments. In total, 32 of 78 (41%) participants added free‐text comments, which are summarized in Table 1.

**TABLE 3:** Rating of a CPR Video Decision Aid by pet owners (n = 78 participants except n = 77, where indicated by “†”).

Question to participant	*n*	%
How would you rate the CPR video decision aid?		
Excellent	27	35
Good	45	58
Fair	1	1
Neutral	4	5
Poor	1	1
How would you rate the amount of information in the video?		
Much less than I needed	0	0
A little less than I needed	7	9
About the right amount	68	87
A little more than I needed	3	4
A lot more than I needed	0	0
How balanced was the video information about CPR?		
Clearly supporting CPR	4	5
A little biased towards having CPR	0	0
Completely balanced	66	85
A little slanted towards not having CPR	8	10
Clearly biased towards not having CPR	0	0
How clear was everything in the video?		
Everything was clear	50	64
Most things were clear	24	31
Neutral	4	5
Some things were clear	0	0
Many things were not clear	0	0
How helpful was the video in helping you make decisions about CPR?^†^		
Very helpful	27	35
Somewhat helpful	35	45
Neutral	12	16
Somewhat unhelpful	2	3
Very unhelpful	1	1
Would you recommend this video to other pet owners?		
I would definitely recommend it	45	58
I would probably recommend it	27	35
Neutral	6	8
I would probably not recommend it	0	0
I would definitely not recommend it	0	0

## 4 Discussion

This study aimed to investigate the impact of a CPR‐VDA on pet owner decision making regarding CPR for their pet. A short CPR‐VDA was designed, and study participants were asked to complete questionnaires before and after viewing the CPR‐VDA. Questionnaire results showed that after viewing the CPR‐VDA, pet owners’ knowledge increased and they felt more confident when taking a decision, although still conflicted in the final decision made. Feedback on the CPR‐VDA was positive, with most participants finding it helpful and recommending its use with other pet owners.

There are no consensus guidelines regarding the design of decision aids, but the IPDAS collaboration has published a checklist for users to determine the quality of decision aids, which informed the design of the CPR‐VDA in this study [10]. The key objectives were to present clear, accurate information in an unbiased manner and in sufficient detail for owners to take decisions about resuscitation for their pet. An audiovisual format allowed visual demonstration of interventions involved in CPR such as intubation and chest compressions. Furthermore, visual information has been shown to result in higher comprehension levels than oral or written aids [17]. Pictographs were used to display survival percentages, as these have been shown to be interpreted more accurately than other visual formats for presenting risk information [18]. Efforts were made to ensure accessibility including closed captioning, narration, and adherence to educational guidelines in the preparation of the CPR‐VDA [19]. In retrospect, there was limited audio description of visual content, and this would need to be addressed in future development of veterinary CPR‐VDAs. While the majority of participants felt that the CPR‐VDA was balanced in its approach, a small proportion considered it biased toward or against CPR. Future efforts should be made to limit bias when presenting probabilities and outcomes in future decision aids. The IPDAS checklist suggests that all relevant options (including doing nothing) should be presented in a decision aid. The CPR‐VDA used in this study presented an alternative option of euthanasia in the event of cardiopulmonary arrest, but the option of doing nothing should also be incorporated into any future veterinary CPR‐VDAs. Overall, however, participant evaluation of clarity and lack of bias was very positive.

Increase in knowledge following the video intervention was chosen as a key measure of the clarity of information provided. As expected, there was an increase in knowledge following the video intervention, with participants having a clearer understanding of the definition of CPR, CPR interventions, and survival to discharge rates. The most marked difference related to understanding of survival to discharge, which aligns with the unrealistic survival expectations previously described in a veterinary study [5].

Prior to the intervention, the majority of participants opted for “resuscitation.” Interestingly, most participants did not change their resuscitation decision following the video intervention. This is in contrast to many human studies, which tend to show a decreased preference for life‐sustaining treatment alongside increased knowledge of CPR after watching a CPR‐VDA [20, 21, 22, 23, 24, 25, 26]. However, there is considerable variation in the design and quality of these studies, and some studies show no change in preference [27, 28]. An important limitation of this study is the omission of providing participants with different decision options based on the underlying cause of arrest. Several participants specifically commented that after watching the CPR‐VDA, their resuscitation decision would depend on the underlying cause of arrest. This omission may account for some of the postintervention decisions changing from “resuscitation” to “not sure,” and thus, these results should be interpreted with caution. Inclusion of this detail should be considered for future studies evaluating veterinary CPR‐VDAs. Studies evaluating veterinary survival to discharge statistics after implementation of the 2012 RECOVER guidelines show variable results, with outcomes remaining generally poor [29, 30]. However, one study showed higher survival‐to‐discharge rates of cats following implementation of these guidelines [6]. In the current study, multiple survival statistics were not provided to pet owners in an effort to provide succinct information; however, it is possible that provision of higher survival rates may have influenced pet owners’ resuscitation decisions, and this could be considered in future studies.

Alongside an increase in knowledge, human healthcare studies suggest that people exposed to decision aids may have a better match between values and choices and show an increased confidence and reduced uncertainty in their decisions [2]. In the current study, participants felt significantly more confident in their resuscitation decision after viewing the CPR‐VDA. However, the degree of increase in confidence was relatively small, with the main increase being in the group feeling “somewhat confident” and an unchanged number feeling “extremely confident.” Despite the increase in confidence, decisional conflict was similar before and after the video intervention. The reason for this is unclear, although the omission of different decision options depending on the cause of arrest in the postintervention questionnaire may play a part. Further investigation of pet owner confidence and uncertainty in CPR decision making is warranted.

Pet owners also need to find the CPR‐VDA acceptable if it is to be a useful tool. In this study, a relatively large proportion of pet owners approached for recruitment declined to participate, with the majority citing stress or upset as their reason. These emotions may relate to research participation at a time of anxiety about their pet, but it is also possible that the prospect of watching a CPR‐VDA was stressful in and of itself. This is an important consideration when introducing such tools into practice and warrants careful thought on mitigation strategies. In human health care, there is an increasing move toward web‐based formats [31, 32]. Providing access to an online CPR‐VDA via a QR code or other method would give owners control over when and how they watch it, which may help to alleviate associated stress. Overall, however, our results show that those owners that participated found the CPR‐VDA highly acceptable. This related to clarity, balance, and overall rating. Importantly, participants felt the video was helpful for making their CPR decision and were likely to recommend it to other pet owners. Free‐text comments also reflected an overall positive impression, with a number of participants explicitly commenting that they found the CPR‐VDA informative. These results are similar to those found in human studies, where patient acceptance of CPR‐VDAs tends to be strongly positive [33, 34].

Pet owner demographics were collected because sociocultural factors such as patient age, gender, religion, and level of education have been shown to influence patients’ healthcare decisions and communication with healthcare providers in human medicine [35, 36, 37]. In contrast, in this study, there was no association between age, education, or religious beliefs and any of initial CPR preference, change in CPR preference, or pre‐knowledge of CPR. The overall educational level of participants was relatively high, and the proportion of owners who felt religion was very important to them was very low. This may have affected results and should be considered when developing CPR‐VDA tools in different demographic areas. Data were not collected on participants’ medical training or previous knowledge of CPR. Furthermore, a small number of participants had previously discussed CPR for their pet, and it is possible that this could have improved their initial knowledge base. While this could have biased the data, the expected impact would be to reduce the difference in pre‐ and postintervention knowledge. Given that knowledge improved, the impact, if any, was likely small.

This study only assessed pet owners presenting through an Emergency & Critical Care Service at a referral hospital. This decision was made for ease of hospital workflow, with study recruitment and intervention taking place during initial triage and stabilization of the pet. However, emergency patients are more likely to be critically ill than patients presenting for a routine referral appointment (e.g., to an orthopedic surgery service), and the severity of their pet's illness may have affected owners’ resuscitation decisions. CPR decisions are important in both groups of patients, and investigating the use of CPR‐VDAs in a variety of patient populations and comparing pet illness severity with pet owner preferences may be useful in future studies.

The aim of this study was to evaluate the acceptability of a CPR‐VDA in veterinary patients and to assess its effect on knowledge and decision making. Thus, a single interventional group was recruited. However, the relative effects of using a CPR‐VDA versus audio‐only or written‐only decision aids or standardized discussions with clinicians were not assessed. This would be interesting to explore further, and the inclusion of comparative groups may be worth considering in future studies. It may also be useful to assess the use of a web‐based CPR‐VDA, which could increase flexibility for owners as described above, in particular when some family members are unable to be present at the consultation.

The clinical relevance of this study and future similar studies should not be underestimated. As stated earlier, 28% of participants changed their decision following the video, 79% of participants described the video as “very or somewhat helpful,” and 92% stated they would recommend it to other pet owners. These findings suggest that effective client education on CPR is tantamount to informed decision making on admission. It is recognized that a CPR‐VDA tool may not be applicable in all situations and that in the case of unstable patients who are admitted under time‐critical conditions, this may not be an appropriate method of education. However, this study confirms the findings of previous studies that there is a knowledge gap among pet owners, which may have profound implications for both patients and the clinical staff providing CPR [5, 38]. Moreover, it suggests that this may impact pet owner decisions on CPR for their pet. CPR is not a benign intervention, with potential for both physical and (in people) psychological harm to patients [37, 39, 40]. This should not be ignored when obtaining informed consent for CPR. In addition, the ethical implications for those patients for whom long‐term prognosis is poor should be considered and relayed to owners effectively. In general, the CPR‐VDA was well received by this pet owner population, and it positively affected their knowledge and decision‐making confidence. Wider use of decision aids may better educate pet owners and help improve communication and shared decision making between veterinary professionals and pet owners in many aspects of veterinary medicine.

## Author Contributions

**Katherine M. Gane**: conceptualization, data curation, investigation, methodology, writing – original draft, writing – review and editing. **Lotta Wahlden**: data curation, writing – original draft. **Benjamin W. N. Fayers**: data curation, writing – original draft; writing – review and editing. **Tim H. Sparks**: formal analysis, writing – review and editing. **Emily Thomas**: data curation, investigation, methodology, project administration, supervision, writing – original draft, writing – review and editing.

## Ethics Statement

The authors confirm that the ethical policies of the Journal, as noted on the Journal's author guidelines page, have been adhered to and the appropriate ethical review committee approval has been received via the Royal College of Veterinary Surgeons Ethical Review Panel.

## Conflicts of Interest

The authors declare no conflicts of interest.

## Supporting information




**Supporting File 1**: vec70070‐sup‐0001‐SuppMat.docx

## Endnotes

1 Microsoft Forms, Redmond, WA: Microsoft; 2016.

2 Microsoft Excel, Redmond, WA: Microsoft; 2018.

3 Minitab statistical software, Version 21.4.2, State College, PA: Minitab LLC; 2021.
