# Supplementary material for: Evaluation of a Cardiopulmonary Resuscitation Video Decision Aid for Pet Owners
Source: J Vet Emerg Crit Care (San Antonio). 2025 Dec 12;35(6):656–64. doi: 10.1111/vec.70070 (PMC12779167; doi:10.1111/vec.70070)
Supplement: Supplementary file 1 — Supporting File 1: vec70070‐sup‐0001‐SuppMat.docx [file VEC-35-656-s001.docx]

Section 1 - Pet Owner Demographics

What is your age in years? (Please leave blank if you’d prefer not to say)

What gender do you identify with

- Male
- Female
- Other
- Prefer not to say

What is the highest level of education you have completed? *If currently enrolled, highest degree received* to date

- No schooling completed
- Secondary School
- Vocational Qualification
- Postgraduate degree
- Other
- Prefer not to say

How important is religion to you?

1 = not at all important, 5 = very important

Have you previously discussed cardiopulmonary resuscitation (CPR) for this pet?

- Yes
- No
- Not sure

Section 2 - CPR preferences

We routinely ask owners of pets presenting to our hospital as an emergency whether they would like us to attempt resuscitation in the unfortunate event that their pet’s heart and breathing stop.

What is your preference for your pet in this situation?

- Attempt resuscitation
- Do not attempt resuscitation
- Not sure

How confident do you feel making a decision about CPR for your pet?

- Extremely confident
- Somewhat confident
- Neutral
- Somewhat not confident
- Extremely not confident

When asked to make a choice regarding resuscitation of your pet, how conflicted do you feel?

- 1 = no conflict, 5 = high conflict

Section 3 - Knowledge Assessment

Question 1 – True or False: CPR is a medical procedure that is performed on pets whose heart has stopped beating in an attempt to restart their heart.

- True
- False
- Not sure

Question 2 – True or False: CPR success rates in dogs and cats are similar to those in humans.

- True
- False
- Not sure

Question 3 - Please select all that apply: CPR can involve:

- Passing a tube down the patient’s airway
- Pressing hard and fast on the chest to pump blood round the body
- Giving medications to restart the heart
- Giving electric shocks to restart the heart
- Not sure

Question 4 – when a patient’s heart stops beating, brain death will occur

- In several minutes
- In several hours
- In several days
- Not sure

Question 5 – If 100 dogs & cats have a chronic condition (e.g. heart failure, kidney failure) and their heart stops, how many will survive CPR and recover well enough to leave the hospital?

- None survive
- Only a small number survive
- Almost half survive
- Almost all survive
- Not sure

Question 6 – if 100 dogs & cats have an anaesthetic drug related complication and their heart stops, how many will survive CPR and recover well enough to leave the hospital?

- None
- Only a small number survive
- Almost half survive
- Almost all survive
- Not sure

Question 7 - True or False: If my pet receives CPR due to an underlying health condition (e.g. heart failure), he/she will still require ongoing care for their illness following successful CPR.

- True
- False
- Not sure

Question 8 - Please pick one answer - If a pet owner decides not to have CPR performed on their pet:

- They will not be offered any other treatments
- Other options for treatment to relieve suffering and discomfort will be offered

Question 9 - Please pick one answer - The veterinary team want to speak to pet owners about CPR decisions because the decision about CPR:

- relies only on the pet's medical condition
- relies only on what is important to the pet owner
- depends on what is most important to the pet owner in addition to the pets medical condition

Section 4

You will now be asked to watch a short video on CPR

Please watch the video and press ‘next’ to progress

Section 5 - CPR Preferences (Post Video)

We routinely ask owners of pets presenting to our hospital as an emergency whether they would like us to attempt resuscitation in the unfortunate event that their pet’s heart and breathing stop.

What is your preference for your pet in this situation?

- Attempt resuscitation
- Do not attempt resuscitation
- Not sure

How confident do you feel making a decision about CPR for your pet?

- Extremely confident
- Somewhat confident
- Neutral
- Somewhat not confident
- Extremely not confident

When asked to make a choice regarding resuscitation of your pet, how conflicted do you feel?

- 1 = no conflict, 5 = high conflict

Section 6 - Knowledge Assessment (Post Video)

Question 1 – True or False: CPR is a medical procedure that is performed on pets whose heart has stopped beating in an attempt to restart their heart.

- True
- False
- Not sure

Question 2 – True or False: CPR success rates in dogs and cats are similar to those in humans.

- True
- False
- Not sure

Question 3 - Please select all that apply: CPR can involve:

- Passing a tube down the patient’s airway
- Pressing hard and fast on the chest to pump blood round the body
- Giving medications to restart the heart
- Giving electric shocks to restart the heart
- Not sure

Question 4 – when a patient’s heart stops beating, brain death will occur

- In several minutes
- In several hours
- In several days
- Not sure

Question 5 – If 100 dogs & cats have a chronic condition (e.g. heart failure, kidney failure) and their heart stops, how many will survive CPR and recover well enough to leave the hospital?

- None survive
- Only a small number survive
- Almost half survive
- Almost all survive
- Not sure

Question 6 – if 100 dogs & cats have an anaesthetic or drug overdose and their heart stops, how many will survive CPR and recover well enough to leave the hospital?

- None
- Only a small number survive
- Almost half survive
- Almost all survive
- Not sure

Question 7 - True or False: If my pet receives CPR due to an underlying health condition (e.g. heart failure), he/she will still require ongoing care for their illness following successful CPR.

- True
- False
- Not sure

Question 8 - Please pick one answer - If a pet owner decides not to have CPR performed on their pet:

- They will not be offered any other treatments
- Other options for treatment to relieve suffering and discomfort will be offered

Question 9 - Please pick one answer - The veterinary team want to speak to pet owners about CPR decisions because the decision about CPR:

- relies only on the pet's medical condition
- relies only on what is important to the pet owner
- depends on what is most important to the pet owner in addition to the pets medical condition

Section 7 - Acceptability of CPR-VDA

Please answer the following questions based on the video you have just viewed.

How would you rate the CPR video decision aid?

- Excellent
- Good
- Neutral
- Fair
- Poor

How would you rate the amount of information in the video?

- Much less than I needed
- A little less than I needed
- About the right amount
- A little more than I needed
- A lot more than I needed

How balanced was the video information about CPR?

- Clearly supporting CPR
- A little biased towards having CPR
- Completely balanced
- A little slanted towards not having CPR
- Clearly biased towards not having CPR

How clear was everything in the video?

- Everything was clear
- Most things were clear
- Neutral
- Some things were clear
- Many things were not clear

How helpful was the video in helping you make decisions about CPR?

- Very helpful
- Somewhat helpful
- Neutral
- Somewhat unhelpful
- Very unhelpful

Would you recommend this video to other pet owners?

- I would definitely recommend it
- I would probably recommend it
- Neutral
- I would probably not recommend it
- I would definitely not recommend it

Did you experience any problems or distractions while viewing the video?

- ___________________

Please leave any comments you may have about the video or survey if you wish

- ___________________

**Section 8**

**Thank you very much for taking the time to complete this questionnaire.**

**If you have any questions, please ask your study investigator.**
